# Estudio comparativo de un nuevo dispositivo para el análisis de la calidad seminal y la evaluación microscópica manual en un laboratorio clínico no especializado

**DOI:** 10.1515/almed-2024-0182

**Published:** 2024-11-26

**Authors:** Erika Jani, Margherita Bozzola, Elmar Marco Zagler, Vincenzo Roccaforte, Massimo Daves

**Affiliations:** Laboratorio de Bioquímica Clínica, Hospital Provincial de Bolzano (SABES-ASDAA), Bolzano, Italia; Laboratorio de Patología Clínica, ASST Norte de Milán, Sesta San Giovanni, Italia

**Keywords:** fertilidad, estandarización, comparación, semen

## Abstract

**Objetivos:**

En el análisis de líquido seminal se miden una serie de parámetros del semen humano con gran relevancia para los estudios de fertilidad, confirmación de esterilidad tras la vasectomía, el seguimiento de patologías como el varicocele y en aquellos casos en los que se precise conservar el esperma. El examen manual del líquido seminal se caracteriza por una baja reproducibilidad. El objetivo del presente estudio es evaluar la eficacia de un instrumento automático para el análisis del semen comparando los resultados con los obtenidos mediante microscopía manual.

**Métodos:**

Se analizaron 50 muestras (edad 18-59 años) aplicando el método manual y el método automatizado simultáneamente. El análisis manual fue realizado por al menos dos analistas experimentados. La concentración y la motilidad se determinaron mediante análisis manual convencional, así como con el analizador automático LensHooke™, siguiendo la guía más reciente de la OMS.

**Resultados:**

Comparamos la concentración (millones/mL) de espermatozoides obtenida mediante cuantificación manual y automática, aplicando la siguiente clasificación: muestras normales, oligospérmicas, criptospérmicas y azoospérmicas. La prueba de Wilcoxon no mostró diferencias estadísticamente significativas. El diagrama de Bland-Altman arrojó un valor levemente superior en la cuantificación manual. Posteriormente, comparamos la morfología y se clasifican las muestras en morfológicamente normales y anormales. A continuación, comparamos la motilidad de los espermatozoides establecida mediante recuento manual y automático, clasificando los resultados como motilidad total normal y astenozoospermia. El análisis estadístico mostró un grado de concordancia moderado en la morfología y muy bueno en la motilidad.

**Conclusiones:**

Los resultados de este estudio demuestran que el dispositivo LensHooke™ posee un nivel de concordancia aceptable con el análisis de líquido seminal mediante microscopía manual. El uso de esta sencilla herramienta podría ayudar a estandarizar los informes de resultados de los laboratorios no especializados.

## Introducción

El análisis de semen es un estudio diagnóstico cuantitativo y cualitativo que permite investigar diferentes parámetros del semen humano de gran relevancia para los estudios de fertilidad, la confirmación de esterilidad tras la vasectomía, el seguimiento de patologías como el varicocele y en aquellos casos en los que se precise conservación de esperma. Actualmente, el análisis manual del semen (AMS) es el método estándar en la evaluación de la fertilidad masculina [1].

El AMS consiste en una evaluación macroscópica y microscópica. En la evaluación macroscópica se examinan el volumen, aspecto y color del líquido seminal, así como algunas características reológicas como la viscosidad y la licuefacción. En la evaluación microscópica, se examinan la concentración, motilidad y morfología de los espermatozoides. El AMS es esencial a la hora de determinar la fertilidad masculina, aunque para una evaluación completa es necesario revisar la historia clínica, así como realizar otras pruebas analíticas (p.ej. análisis hormonales) y estudios de imagen [2].

De este modo, en la práctica clínica, el análisis de la calidad seminal es la prueba diagnóstica más frecuente y fundamental en el manejo de las parejas subfértiles, pudiendo orientar al clínico sobre los estudios adicionales a realizar en la evaluación de la infertilidad 3], [4], [5.

En las últimas décadas, el límite inferior de referencia para el recuento de espermatozoides se ha ido rebajando paralelamente al permanente debate sobre la disminución global de la calidad seminal, con resultados contradictorios y ningún consenso resultante, por tanto. La OMS intentó establecer valores de referencia para los parámetros seminales, fundamentados en varios estudios retrospectivos y prospectivos realizados en diferentes países. En la última edición del manual de la OMS para el análisis del semen, se propuso como novedad el percentil 5 como el límite inferior de normalidad. Dicho valor se deriva de la distribución de valores obtenidos en hombres que lograron una concepción natural en un periodo inferior a doce meses [1].

El AMS, como la mayoría de exámenes diagnósticos realizados de forma manual, se caracteriza por una baja reproducibilidad, debido a la interpretación subjetiva que estos implican, lo cual puede afectar a la correcta clasificación de la calidad seminal. Por otro lado, el AMS es un procedimiento laborioso que requiere la intervención de analistas debidamente cualificados y experimentados [3]. Para superar las limitaciones del procesamiento manual, sería necesario disponer de un dispositivo automático para la evaluación de la calidad seminal. El objetivo del presente estudio es evaluar la eficacia de un analizador automático de calidad seminal, comparando sus resultados con los obtenidos mediante microscopía manual.

## Materiales y métodos

Se analizaron 50 muestras (edad 18–59 años) simultáneamente con el microscopio manual y con el analizador automático durante un periodo de 25 días consecutivos (dos muestras al día). El análisis manual fue realizado por al menos dos analistas experimentados. Las muestras se recogieron tras un periodo de 2 a 7 días de abstinencia, siendo estas enviadas al laboratorio en los 45 minutos posteriores a la masturbación. Los parámetros seminales, entre los que se incluyen la concentración, motilidad y pH seminal, se determinaron mediante el análisis manual estándar del semen, así como empleando el analizador automático de calidad seminal LensHooke™ X1 PRO, siguiendo las directrices de la 6^a^ edición de la OMS (2021). Se evaluaron dos muestras seminales distintas al día, durante 25 días laborables consecutivos. La concentración de esperma (× 10^6^/mL) se evaluó empleando una cámara de recuento Makler, tras la licuefacción de la muestra seminal en el termostato a 37 °C, durante un periodo de entre 30 y 60 minutos. La motilidad espermática (%) se evaluó a temperatura ambiente, cuantificando al menos 100 espermatozoides en el microscopio óptico a una amplificación de 250 × (esto es, la combinación de un lente objetivo de 25 × con una ocular de 10 ×). Los espermatozoides se clasificaron en cuatro categorías: progresivos rápidos, progresivos lentos, no progresivos e inmóviles, tal como se recomienda en la sexta edición del manual para la evaluación y procesamiento del semen humano de la OMS. La morfología se evaluó con el microscopio de contrate de fases a la amplificación máxima de 400 × , combinación de un objetivo de 40 × y un ocular de 10 ×.

Se realizó un análisis seminal automático con el analizador LensHooke™ X1 PRO Semen Quality Analyzer (Bonraybio Co., Ltd, Taichung City, Taiwan) para medir la concentración y motilidad del esperma, así como el pH seminal, con ajuste a las instrucciones de la 6^a^ Edición de la OMS (OMS, 2021) y a las instrucciones del fabricante.

El analizador de calidad seminal LensHooke^®^ X1 PRO, empleado conjuntamente con el test en formato *cassette* LensHooke^®^ (Bonraybio Co., LTD), es un dispositivo óptico para el análisis de semen humano, que proporciona las medidas cuantitativas directa y calculada de concentración y motilidad del esperma (porcentaje de motilidad progresiva, porcentaje de motilidad no progresiva, porcentaje de inmovilidad), morfología espermática (porcentaje con morfología normal) y pH [6]. Este instrumento utiliza un microscopio óptico de alta resolución con autofocus, en combinación con un sistema de interpretación basado en el uso de inteligencia artificial [3, 6]. Se aplicó la prueba de Wilcoxon para evaluar la presencia de diferencias estadísticamente significativas entre los resultados obtenidos con los dos métodos. Se estableció un valor p<0,05 para detectar diferencias estadísticamente significativas. El nivel de concordancia entre los resultados de concentración espermática y motilidad total obtenidos con los dos métodos se analizó con el método de Bland-Altman y el análisis de regresión de Passing y Bablok. Por otro lado, el nivel de concordancia cualitativa entre valores categóricos se evaluó mediante el coeficiente kappa ponderado. Todos los análisis estadísticos se realizaron con el programa MedCalc17.4.4^©^ statistical (MedCalc Software, Ostend, Bélgica).

El estudio se realizó con ajuste a la versión más reciente de los principios de la Declaración de Helsinki establecidos por la Asociación Médica Mundial. No se consideró necesario solicitar aprobación formal por parte del Comité de Ética, ya que la evaluación de los seminogramas formaba parte de las pruebas ordinarias prescritas a los pacientes, el estudio no interfirió en la práctica clínica habitual, y la totalidad de los resultados fueron anonimizados, previamente a su evaluación.

## Resultados

**Figura 1:**
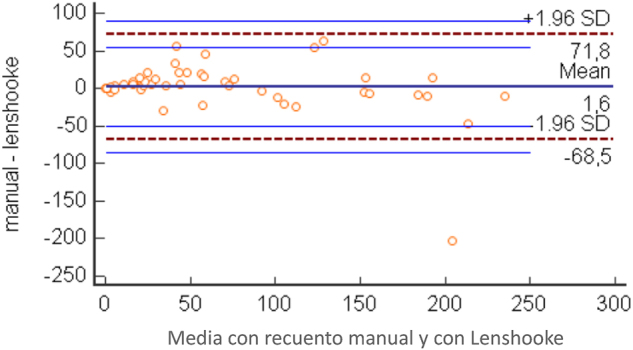
Diagrama de Bland-Altmann en el que se compara la concentración espermática obtenida mediante recuento manual y automático. Diferencia media: 1,6 millones/mL.

Inicialmente, se comparó la concentración de espermatozoides (millones/mL) obtenidos mediante recuento manual y automático. La prueba de D’Agostino-Pearson descartó la normalidad en la distribución de los datos para ambos métodos de recuento. El valor de la mediana obtenido con el recuento manual fue de 50,5 millones/mL, con un intervalo de confianza (IC) del 95 % para la mediana de 27,60 a 75, (valor máximo 230 millones, valor mínimo 0), rango intercuartílico (RI) de 20 a 95 millones/mL. Con el instrumento de LensHooke, se obtuvo una mediana de 35 millones/mL, IC 95 % de 19,9 a 66,27 (valor máximo 306 millones, valor mínimo 0,3), RI 13,4–97,3. La regresión de Passing y Bablok fue Y= −4,03 + 1,01X, (ordenada en el origen −4,03; IC95 %: de −7,31 a −0,47; pendiente 1,01, IC95 %: 0,89-1,09) donde Y= instrumento de Lenshooke y X= recuento manual. Coeficiente de correlación de Spearman 0,944. La prueba de Wilcoxon no mostró diferencias estadísticamente significativas. El diagrama de Bland-Altman, en el que se comparó el recuento manual y el instrumental mostró un leve sesgo positivo, indicando un valor ligeramente superior para el recuento manual (Figura 1).

Así mismo, comparamos las diferentes clasificaciones obtenidas con los dos métodos de recuento en muestras normales (concentración espermática >16 millones/mL), oligospérmicas (<15 millones/mL), criptospérmicas (concentración espermática <1 millones/mL) y azoospérmicas (sin espermatozoides). En la Tabla 1 se muestran los resultados; el índice kappa ponderado fue de 0,761 (IC95 % de −0,585 a 1,000) lo que refleja un buen nivel de concordancia. Cabe señalar que esta nomenclatura desaparece en la 5^a^ edición, con la introducción del término *“límite de decisión”* para describir la desviación de los valores de referencia para el semen. Posteriormente, comparamos la morfología y clasificación de las diferentes muestras en muestras morfológicamente normales y anormales (teratozoospermia: espermatozoides morfológicamente anormales >96 %). En la Tabla 2 se muestran las diferentes clasificaciones en función de la morfología, con un índice kappa ponderado de 0,52 (IC95 %, de −1.000 a 1.000), presentando un nivel de concordancia moderado.

**Tabla 1:** Diferencias en la clasificación de las muestras según la concentración espermática; kappa ponderado: 0,761.

	Recuento manual				
Lenshooke	Azo	Cripto	Oligo	Normo	
Azo	0	0	0	0	0
Cripto	0	1	0	0	1 (2 %)
Oligo	0	0	0	38	38 (76 %)
Normo	1	1	9	0	11 (22 %)
	1 (2 %)	2 (4 %)	9 (18 %)	38 (76 %)	50

Azo, azoospermia; Cripto, criptozoospermia; Oligo, oligozoospermia; Normo, normozoospermia.

**Tabla 2:** Diferencias en la clasificación de las muestras según la morfología espermática; kappa ponderado: 0,520.

	Recuento manual		
Lenshooke	Normal	Terato	
Normal	28	1	29 (58 %)
Terato	10	11	21 (42 %)
	38 (76 %)	12 (4 %)	50

Terato, teratozoospermia.

A continuación, comparamos la motilidad de los espermatozoides obtenida con el recuento manual y automático, clasificando los resultados como motilidad total normal y astenozoospermia. Según las indicaciones más recientes de la OMS, para que la motilidad sea considerada normal en una muestra, al menos un 42 % de los espermatozoides deben ser móviles. Con respecto a la motilidad total (motilidad progresiva rápida, motilidad progresiva lenta, motilidad no progresiva, expresada como porcentaje con respecto al número total de espermatozoides), la mediana obtenida mediante recuento manual fue del 55,5 %, IC95 % para la mediana de 48,21 a 68,59, valor máximo 90 %, valor mínimo 0 %), RI de 37 a 77 %. Con el instrumento de LensHooke, se observó una mediana de 60,5 %, IC95 % de 41,21 a 72 (valor máximo 99 %, valor mínimo 3 %), RI de 30 a 76. La prueba de Wilcoxon no mostró diferencias estadísticamente significativas. La regresión de Passing y Bablok fue Y=2.64+1.04X, donde Y=instrumento de Lenshook y X= recuento manual, ordenada en el origen 2,64 (IC95 %: de −13,26 a 11,24), pendiente 1,04 (IC95 %: de 0,84 a 1,35). Coeficiente de correlación de Spearman 0,688. En la Tabla 3 se muestran las diferentes clasificaciones en función de la motilidad total con un índice kappa ponderado de 0,839 (IC95 % de −1,000 a 1,000), lo que refleja un muy buen nivel de concordancia.

**Tabla 3:** Diferencias en la clasificación de las muestras según la motilidad total de los espermatozoides; kappa ponderado: 0,839.

	Recuento manual		
Lenshooke	Asteno	Normal	
Asteno	14	0	14 (28 %)
Normal	4	32	36 (42 %)
	18 (36 %)	32 (64 %)	50

Asteno, astenozoospermia.

## Discusión

El manual de laboratorio de la OMS para el examen y el procesamiento del semen humano es considerado el manual de referencia para los laboratorios que realizan análisis de líquido seminal. Sin embargo, la interpretación y aplicación de los anteriores valores “normales” o “de referencia” de la OMS para los parámetros seminales presentan algunas limitaciones, ya que los datos se obtuvieron de poblaciones de referencia insuficientemente definidas y fueron obtenidos en distintos laboratorios con diferentes métodos analíticos [7].

Resulta muy difícil establecer parámetros seminales individuales que predigan la probabilidad de embarazo, incluso en el contexto de la reproducción asistida. Esto se puede explicar por la falta de estandarización de los seminogramas, debido al uso de metodologías diferentes y a los diferentes niveles de experiencia de los analistas [8, 9].

Las principales limitaciones a la hora de estandarizar el AMS son la subjetividad en la interpretación de los resultados y su dependencia de la pericia del analista. Esta última resulta muy relevante en el laboratorio clínico general, donde el análisis de líquido seminal únicamente representa una pequeña parte de la actividad del laboratorio, frente a los laboratorios especializados en el diagnóstico de la infertilidad. La introducción de un instrumento que garantice una mayor estandarización de los seminogramas supone una mejora en la práctica del laboratorio, especialmente para los laboratorios no especializados. En el presente estudio, evaluamos los resultados obtenidos con el analizador LensHooke X1 en el análisis de la calidad espermática, comparados con el análisis mediante microscopía manual tradicional. El aspecto más importante a analizar fue la capacidad del instrumento para clasificar a los pacientes en función de la concentración, morfología y motilidad de los espermatozoides, en concordancia con la clasificación obtenida mediante microscopía manual. Los resultados de nuestro estudio coinciden con los de otros estudios previos, que concluyeron que los datos de concentración y motilidad espermática obtenidos con el analizador LensHooke son comparables a los obtenidos con la evaluación microscópica manual [5]. Para la concentración espermática, el diagrama de Bland-Altman en el que se compara el recuento manual con el instrumental mostró un ligero sesgo positivo, indicando un valor levemente superior para el recuento manual, corroborado por la regresión de Passing-Bablok, que demuestra además la ausencia de un sesgo proporcional (Y=−4,03 + 1,01X; ordenada en el origen −4,03; IC95 %: de −7,31 a −0,47; pendiente 1,01, IC95 %: 0,89-1,09) donde Y= instrumento de Lenshooke y X= recuento manual). La comparación entre el método manual y el método instrumental en los resultados de motilidad espermática, así como en la clasificación de motilidad total normal a astenozoospermia, resulta más que aceptable, tal como muestra el índice kappa ponderado de 0,839. Así mismo, evaluamos el grado de concordancia entre estos dos métodos en el examen morfológico, considerando que la distinción entre espermatozoides morfológicamente normales y anormales seguirá siendo un factor relevante a la hora de evaluar la función espermática humana en la práctica clínica [8, 9]. Obtuvimos un índice kappa ponderado de 0,52, que representa un nivel de concordancia moderado, lo cual no es de extrañar, teniendo en cuenta las considerables diferencias metodológicas entre la evaluación por microscopía y por análisis automatizado: la primera se basa en la observación visual por parte del analista, por lo que la interpretación depende de su experiencia la segunda se basa en un algoritmo preciso, que analiza de forma cruzada la medida de las dimensiones de la cabeza y la cola de los espermatozoides y los clasifica. En todo caso, el grado de concordancia que obtuvimos parece ser aceptable para una evaluación inicial de la calidad seminal en el laboratorio no especializado en la evaluación de parejas infértiles. Además, las pequeñas dimensiones del instrumento y su facilidad de uso son apropiadas para cubrir las necesidades del laboratorio no especializado. Además de una mayor sensibilidad, otra de las ventajas que ofrece esta técnica es la facilidad de evaluación e interpretación de resultados con respecto a la evaluación con el microscopio óptico.

El presente estudio presenta ciertas limitaciones que deben señalarse. En primer lugar, el reducido número de muestras empleadas para la comparación. Ciertamente, sería necesario realizar más estudios con un mayor número de muestras para determinar la correlación entre el método manual y el automático. Otra limitación importante es que no empleamos la tinción recomendada por la guía más reciente de la OMS para la evaluación de la morfología, sino la observación con el microscopio de contraste de fases. Este procedimiento no está en consonancia con las últimas recomendaciones de la OMS para la evaluación de la morfología de los espermatozoides, que requiere la preparación de un frotis de eyaculado en un portaobjetos, y la fijación y tinción con la tinción de Papanicolau [1, 5]. El uso eventual de otra tinción debería ser validado con respecto a la tinción Papanicolau de referencia [1, 5]. En nuestro laboratorio, comparamos recientemente la morfología obtenida mediante contraste de fases con la obtenida mediante la tinción en portaobjetos, lo que nos permite seguir empleando el microscopio de contraste de fases, según las antiguas recomendaciones. Otra limitación fue que no empleamos muestras post-vasectomía para determinar si, en estos casos, LensHooke™ X1 PRO es capaz de detectar o no espermatozoides.

En conclusión, el analizador LensHooke™ X1 PRO muestra un aceptable nivel de concordancia con la evaluación manual seminal y es además una técnica fácil de implementar. Estas características son favorables a una más amplia utilización de esta instrumentación en la práctica clínica.

El uso de esta sencilla herramienta podría ayudar a estandarizar los informes y la información clínica de los laboratorios de primer nivel, no especializados en el análisis de calidad seminal.
